# The Impact of Different Coping Styles on Psychological Distress during the COVID-19: The Mediating Role of Perceived Stress

**DOI:** 10.3390/ijerph182010947

**Published:** 2021-10-18

**Authors:** Yi Ding, Xinchen Fu, Rude Liu, Jacqueline Hwang, Wei Hong, Jia Wang

**Affiliations:** 1 Graduate School of Education, Fordham University, New York, NY 10023, USA; yding4@fordham.edu (Y.D.); jhwang26@fordham.edu (J.H.); 2 Beijing Key Laboratory of Applied Experimental Psychology, National Demonstration Center for Experimental Psychology Education, Faculty of Psychology, Beijing Normal University, Beijing 100875, China; fxc_psy@163.com (X.F.); psyhongwei@163.com (W.H.); 3 Teachers’ College, Beijing Union University, Beijing 100874, China; wangjia@mail.bnu.edu.cn

**Keywords:** coping styles, psychological distress, mediating role, perceived stress, COVID-19

## Abstract

The present study aimed to understand the impact of different coping methods endorsed by Chinese college students during COVID-19 through the examination of the mediating role of perceived stress. We recruited a total of 492 undergraduate students to complete an online survey from May to June 2020. The results of structural equation modeling indicated that perceived stress was a significant mediator in the association between different coping styles and psychological distress. Three coping styles, including problem-focused, adaptive emotion-focused, and maladaptive emotion-focused coping styles were all significantly correlated with psychological distress. Perceived stress significantly mediated the association between the three coping styles and psychological distress. The results indicated a full mediation model in which problem-focused coping and adaptive emotion-focused coping affected psychological distress entirely through the mediation of perceived stress. Maladaptive emotion-focused coping positively predicted perceived stress, which in turn positively predicted psychological distress through a partial mediation model. We discuss the implications of these findings and offer suggestions for future research.

## 1. Introduction

The novel coronavirus behind the current pandemic and disruption of life was given the name of “SARS-CoV-2” and the disease was also named “coronavirus disease 2019” (COVID-19; Centers for Disease Control and Prevention [CDC]) [1]. Wuhan, in the Hubei province of China, was the first city that reported the first case of COVID-19 in late December 2019. Quickly, the virus spread beyond Hubei Province, and Chinese authorities segregated the affected regions and immediately implemented nationwide mitigation measures according to the severity of the reported cases of COVID-19 [2]. Although local transmission of the virus was under control by April 2020 in China, COVID-19 had already spread worldwide. The World Health Organization (WHO) characterized COVID-19 as a pandemic (CDC) [3] on 11 March 2020.

University students have faced numerous challenges during the pandemic. In China, many universities suspended in-person classes and activities in Spring 2021 and through the summer session. Study abroad programs were cut short with universities asking their students to return home. Many university residence halls closed, giving students only a few days’ notice to vacate their dorm rooms [4,5,6]. Some academic disciplines did not allow for efficient online courses. Fieldwork, internships, and clinical rotations were suspended. Given the uncertainty of when life would return to normal [7], students felt uneasy about the course of their academic careers. Due to the effects of COVID-19, many students faced unprecedented stress regarding their living situations, financial burdens, graduation challenges, and job searches [8]. The present study aimed to examine the mediating role of perceived stress on the associations between three different coping styles and psychological distress during the COVID-19 pandemic through the transactional mode of stress and coping proposed by Lazarus and Folkman [9].

### 1.1. Coping Styles

Based on the transactional model of stress and coping developed by Lazarus and Folkman, personal and situational factors influence how one perceives and evaluates encountered situations. Variables within a person and the environment (e.g., beliefs, demands, and constraints) affect stress appraisal and coping strategy use, resulting in physiological and emotional reactions [9]. Coping could be described as humans’ cognitive and/or behavioral efforts that are used to cope with external and internal demands under a stressful circumstance [9,10].

There are different types of coping strategies. Emotion-focused coping is reactive and refers to attempting to regulate feelings and emotional responses to the stressor (e.g., anger, fear, sadness, anxiety, pressure). Problem-focused coping is proactive and refers to acting on the stressor, the environment, or oneself to address the problem in an attempt to decrease or eliminate the stress [10]. It was reported that it is more effective to use problem-focused coping in controllable stressful circumstances, but it is more effective to use emotion-focused coping in uncontrollable stressful circumstances [11,12]. A third type of coping, avoidance-focused coping, refers to cognitions and behaviors aimed at avoiding the stressful situation and reactions to it, such as distraction and diversion, and tends to be an initial reaction to stress [11,13,14,15].

In a study by Kumanova and Karastoyanov that investigated the associations between perceived stress and coping strategies, results showed that people who more often use specific proactive coping strategies, such as reflective coping and strategic planning; specific reactive problem-focused coping strategies, such as effective coping and planning ahead of time; and specific reactive emotion-focused strategies, such as reinterpretation from a positive perspective and growth, experience less stress. Conversely, people who more often use specific reactive emotion-focused coping approaches, such as focusing on emotions, denial, seeking emotional social support, and disengaging, experience more stress [16].

Different coping strategies also appear in response to different stressful situations. One study examined the relationship between stressors and coping in college students when the 2003 severe acute respiratory syndrome (SARS) epidemic occurred, and researchers found that the active coping was predictive of life satisfaction and the avoidant coping was predictive of psychological symptoms. The results indicated that all types of coping buffered against negative impacts to health. In situations of uncontrollable, large-scale stressors, such as SARS 2003, any type of coping appears to help reduce stress [12]. In short, there have been mixed findings regarding coping styles and stress.

### 1.2. Psychological Distress

To slow down and contain the spread of COVID-19, many governments around the world have adopted suppression measures, such as lockdowns, quarantining at home, and bans of social gatherings and public events, which can lead to unintended mental health consequences for the public [17]. Brooks and colleagues identified some of the factors that might contribute to psychological distress in relation to these measures [18]. First, ongoing reports of COVID-19 outbreaks in different countries and regions through social media and the press are likely to increase individuals’ anxiety, depression, and fears associated with COVID-19. Second, both valid and invalid information about the negative consequences of COVID-19 might lead to higher levels of anxiety and depression. Third, high-risk individuals, such as the elderly and those with existing medical conditions, might suffer from more severe anxiety and depression.

In China, one of the major suppression measures has been confinement (e.g., staying at home during quarantine, banning of social gatherings). Confinement limits opportunities for social interaction, and it can negatively affect the mental health of vulnerable individuals [19]. Confinement can lead to increased psychological distress due to limited access to extended family and limited contact with people outside of the home [20]. Suppression measures have also altered conditions at work and school for many people. Many college students had to immediately transition from in-person instruction to fully virtual instruction, which dramatically increased the hours spent on teleworking for both academic activities and employment activities [21] and decreased the opportunities for physical activities [22]. Prolonged hours of teleworking and exclusive virtual learning can lead to mental fatigue and burnout [21].

### 1.3. The Mediating Role of Perceived Stress

If an individual perceives that the demands of a situation are beyond their own capabilities to deal with the circumstances, a sense of stress occurs [9]. The concept of perceived stress highlights that although people might experience the same event (e.g., the COVID-19 pandemic), it is their perception of the event that largely determines their stress response. Unprecedented stressors have affected university students as a consequence of the COVID-19 pandemic [5,23]. The degree to which people find a life situation stressful determines the degree of confidence they have in their ability to cope with that stressful situation. When an individual feels the general stressfulness of their life and realizes that their ability to handle such stress might be limited during specific situations, they perceive stress. In particular, perceived stress consists of factors such as feelings about circumstances that are uncontrollable or unpredictable in one’s life, how much change is occurring, and one’s confidence in one’s ability to deal with the presenting challenges [9,24].

Research has found that perceived stress is associated with self-efficacy, that is, high levels of perceived stress predict low levels of self-efficacy. Additionally, individuals who perceive a stressful situation as a challenge or an opportunity to prove themselves utilize coping skills more effectively and are less likely to think negatively [25,26]. Although all university students have been experiencing the same event, the COVID-19 pandemic, and are likely to cope with the pandemic using different coping styles, it is each student’s perception of the situation that dictates their level of stress, which in turn can affect the onset of psychological distresses, such as mental fatigue, anxiety, and depression.

### 1.4. Purpose of the Study

Although some previous studies examined copying styles, psychological distress, and perceived stress in numerous circumstances, none of them were conducted during a long-lasting public health crisis such as the COVID-19 which affected the public not only at the individual level, but also at the societal level. In addition, there is no single theory to fully support our theoretical model, which proposed to examine how and to what degree that the perceived stress might mediate the relations between copying styles and psychological distress. In the present study, the focus was to examine the mediating role of perceived stress in the associations between different coping styles and psychological distress among college students during COVID-19. The following questions were proposed: How do problem-focused coping, adaptive emotion-focused coping, and maladaptive emotion-focused coping affect mental health during the COVID-19 pandemic? Are there other potential mediation factors between three coping styles and mental health, such as psychological distress during the COVID-19 pandemic? The findings might provide insights to public health providers and mental health service providers in terms of how to provide prevention and intervention strategies to the public, especially during a public health crisis.

## 2. Materials and Methods

### 2.1. Participants

This study was approved by Academic Ethics Committee of the Faculty of Psychology at Beijing Normal University. We recruited 492 Chinese students from two colleges in Beijing who completed the online survey between May and June 2020. The participants comprised 196 (39.8%) males and 296 (60.2%) females. Participants had an average age of 19.51 years (SD = 1.516), with an age range from 17 to 29. Because the public has been through multiple waves of the COVID-19 surges, it is important to explain the social context of the time when the study was implemented. The first COVID-19 case was identified in Wuhan, China in December of 2019. Starting on 3 February 2020, the Chinese authorities closed off Wuhan (a city of 11 million) by canceling planes and trains leaving the city, suspending buses, subways and ferries within it and quarantining the non-essential workers for about two months. After that, sporadic quarantine was conducted in different cities in China, based on the number of COVID-19 cases. Thus, in general, the public was under serious pressure due to uncertainty of the virus and the constantly changing policies enforced by the central or local government. During the time period of data collection, no COVID-19 vaccine was available to the public.

### 2.2. Measures

#### 2.2.1. Coping

The measure of coping was adapted from the Brief COPE inventory [27]. This scale initially consisted of 14 subscales and there were two items for each subscale. Yeung and Fung [28] used one item for each subscale, and there were two categories of the items: problem-focused coping and emotion-focused coping. The categories were separated into adaptive emotion-focused coping (e.g., “I’ve been looking for something positive in what is happening”) and maladaptive emotion-focused coping (e.g., “I’ve been using alcohol or other drugs to make myself feel better”). The Brief COPE consisted of 11 items, including three items for problem-focused coping, two items for adaptive emotion-focused coping, and six items for maladaptive emotion-focused coping. Two items that best fit the pandemic situation to measure adaptive emotion-focused coping were chosen, including “I’ve been looking for something good in what is happening” and “I’ve been accepting the reality of the fact which has happened.” Participants were directed to rate their coping during the peak time of COVID-19, ranging from 1 (none) to 5 (always). Cronbach’s α for the measure of coping was 0.794 in the present study. Cronbach’s α for problem-focused coping, adaptive emotion-focused coping, and maladaptive emotion-focused coping were 0.606, 0.823, and 0.772, respectively.

#### 2.2.2. Perceived Stress

The measure for perceived stress was adapted from the Depression Anxiety Stress Scales [29]. The sample items were statements such as “Because of COVID-19, I find it difficult to relax.” Participants rated their perceived stress during the peak time of COVID-19, ranging from 1 (strongly disagree) to 5 (strongly agree). The scale consisted of seven items. Cronbach’s α for perceived stress was 0.905 in the present study.

#### 2.2.3. Psychological Distress

We measured psychological distress using the Chinese version of the 10-item Kessler Scale [30], which was adapted from Kessler et al. [31]. The scale consisted of 10 items. The sample items were statements such as “I felt so sad that nothing could cheer me up.” Participants were asked to rate their relatedness to presented factors during the peak time of COVID-19, ranging from 1 (strongly disagree) to 5 (strongly agree). Cronbach’s α for psychological distress was 0.959 in the present study.

### 2.3. Data Analysis

We used SPSS 19.0 (IBM Corp., Armonk, N.Y., USA) to provide descriptive analyses of the variables, including the means, standard deviations, and Pearson correlations. Mplus 7.1 was used to examine the hypothetical model. We used maximum likelihood (ML) to handle the missing data. We used chi-square values (χ^2^), the comparative fit index (CFI), the Tucker– Lewis fit index (TLI), the root-mean-square error of approximation (RMSEA), and the standardized root-mean-square residual (SRMR) to evaluate the models. In general, an acceptable model fit is indicated by CFI and TLI greater than 0.9 and RMSEA and SRMR less than 0.08.

## 3. Results

### 3.1. Descriptive Statistics and Correlations

This study is all subjective self-reported data. To the validity of the results, we conducted a common method bias test. Harman’s single factor test result showed that the model fit was: χ^2^ = 4275.297, CFI = 0.614, TLI = 0.583, RMSEA = 0.000, SRMR = 0.128. This model was unsatisfactory. Thus, this study did not have serious common method bias. We provide the means, standard deviations, and correlation coefficient in Table 1. Three coping styles, perceived stress, and psychological distress were correlated with each other (r ranging from 0.093 to 0.595). Problem-focused coping was positively correlated with perceived stress and psychological distress. Adaptive emotion-focused coping was negatively correlated with perceived stress and psychological distress. Maladaptive emotion-focused coping was positively correlated with perceived stress and psychological distress.

### 3.2. Examination of the Mediation Model

**Figure 1:**
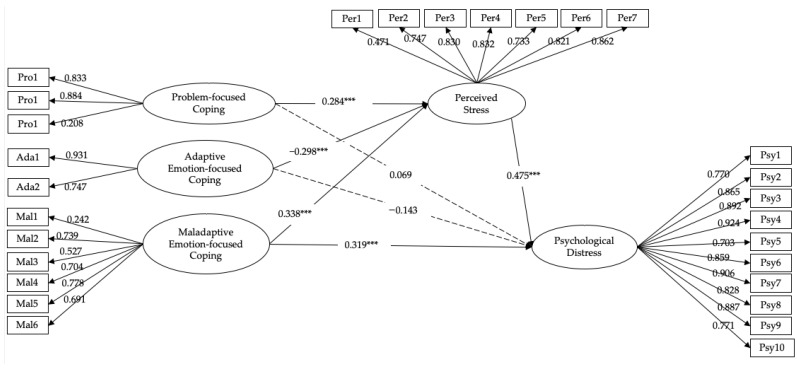
Mediation model of the association between different coping styles and psychological distress. Note: all the loadings on latent variables were significant (*p* < 0.001). ***p < 0.001. Pro = Problem-focused; Ada = Adaptive Emotion-focused Coping; Mal = Maladaptive Emotion-focused Coping; Per = Perceived Stress; Psy = Psychological Distress.

A multiple model (see Figure 1) with the three coping styles as independent variables, perceived stress as the mediator, and psychological distress as the dependent variable was established. The SEM results of the mediation model showed an acceptable model fit: χ^2^/df = 3.337, CFI = 0.923, TLI = 0.913, RMSEA = 0.069, SRMR = 0.077. As shown in Figure 1, maladaptive emotion-focused coping directly and significantly predicted psychological distress. Maladaptive emotion-focused coping positively predicted perceived stress, which in turn positively predicted psychological distress. Similarly, problem-focused coping positively predicted perceived stress, which in turn positively predicted psychological distress. In addition, adaptive emotion-focused coping negatively predicted perceived stress, which in turn positively predicted psychological distress. More importantly, problem-focused coping and adaptive emotion-focused coping did not directly predict psychological distress. The results indicated a full mediation model: problem-focused coping and adaptive emotion-focused coping affected psychological distress entirely through the mediation path.

To further examine whether the indirect effects were significant, we used bias-corrected bootstrap tests derived from 1000 samples. As shown in Table 2, maladaptive emotion-focused coping positively predicted psychological distress, while problem-focused coping and adaptive emotion-focused coping did not predict psychological distress. Perceived stress significantly mediated the association between the three coping styles—problem-focused coping, adaptive emotion-focused coping and maladaptive emotion-focused coping—and psychological distress.

## 4. Discussion

Although previous studies examined the relations between coping styles, psychological distress, and perceived stress in the public, this study examined such relations in the context of the COVID-19 pandemic in Chinese college students in the country where the first case of COVID-19 was officially reported. First, the results showed that three coping styles were all significantly correlated with psychological distress in Chinese college students during the early stage of the COVID-19 pandemic. Adaptive emotion-focused coping was negatively associated with perceived stress and psychological distress. Maladaptive emotion-focused coping was positively associated with perceived stress and distress. These findings concur with those of Kumanova and Karastoyanov [16], suggesting that individuals who use specific reactive emotion-focused coping strategies more often, such as focusing on emotions, denial, seeking emotional social support, and disengaging, experience more stress.

Second, perceived stress significantly mediated the association between problem-focused, adaptive emotion-focused, and maladaptive emotion-focused coping and psychological distress. According to Lazarus and Folkman [9], perceived stress is associated with many psychological factors such as one’s feelings about the unpredictability and uncontrollability of a specific life circumstance, such as the COVID-19 outbreak, and confidence in their abilities to problem solve and cope with the difficulties. Research has suggested that perceived stress can be associated with self-efficacy (i.e., belief about one’s capacities to execute behaviors to achieve certain performance attainments) [24,32]. Previous studies have suggested that individuals who perceived a stressful situation as an opportunity or challenge to prove their abilities tended to utilize their coping skills more effectively and be less likely to have negative thoughts [25,26]. Although the COVID-19 pandemic has had an unprecedented impact on the public, individuals are likely to perceive and interpret the presented situations differently and utilize coping strategies differently, which might contribute to different levels of psychological distress.

Third, problem-focused and adaptive emotion-focused coping affected psychological distress entirely through the mediation path. The findings of the current study suggest that problem-focused and adaptive emotion-focused coping did not directly predict psychological distress. The impacts of problem-focused and adaptive emotion-focused coping on psychological distress were through the mediation role of perceived stress in this study. The associated consequences and effects of the COVID-19 pandemic continue to pose a major challenge to the public. Preventive measures and social distancing requirements have been developed and mandated to contain the spread of the virus and are still ongoing in specific regions [33]. College students have faced a number of challenges, such as sudden closures of university dormitories, cancellation of all in-person instruction and field placements (e.g., practicums and internships), the loss of off-campus jobs that require in-person contact, lack of in-person social support from peers and instructors, and a dramatic reduction in outdoor physical activities [34]. According to Lazarus [35], perceived stress is experienced subjectively by an individual, who might identify an imbalance between the demands placed on them and the available resources to deal such demands. While the rapidly changing societal situations associated with COVID-19 may have appeared to be uncontrollable to some participants, others might have perceived the situation differently, and thus might have subjectively experienced different levels of psychological distress. The findings suggest that mental health providers might want to target strategies and resources that could alleviate perceived stress in the individuals when an uncontrollable pandemic such as the COVID-19 occurs. In other words, individuals might not be able to change the external environment in the context of COVID-19 pandemic, but subjectively changing one’s perception and interpretation of a stressful event might help reduce perceived stress.

The literature has linked exposure to acute stress to both short-term or long-term physical and psychological disorders. Cannon [36] outlined that the human body copes with acute stressors by utilizing emotional and motivational systems. When encountering stressful situations, the human body’s sympathetic nervous system initiates the “fight or flight response,” such as faster heart rate, rapid breathing rate, and excessive sweating. In turn, the parasympathetic responses are diminished to cope with the stressor. Over time, the human body might become exhausted, and such response eventually leads to physical burnout and psychological distress. According to Melamed et al. [37], when an individual is emotionally exhausted and does not have the resources to cope with encountered stressors, psychological burnout and distress might occur. Thus, perceived stress experienced by an individual might directly dictate the pervasiveness and severity of the psychological distress of the individual.

Fourth, different coping styles appear to have differentiated impacts on mental health. Based on the transactional theory, stress can be viewed as an interactive process between the stressors, such as environmental circumstances that negatively affects one’s well-being, and one’s psychological responses, such as appraisal, adjustment, and coping [9]. Based on Lazarus and Folkman (1984), one coping strategy is emotion-focused coping. In the present study, maladaptive emotion-focused coping (e.g., refusal, avoidance, escape, use of alcohol) was separated from adaptive emotion-focused coping (e.g., accepting the reality, or looking for positive aspects in life challenges). Maladaptive emotion-focused coping could lead to passive or avoidant coping. In a stressful situation, it is common that individuals resort to avoidant coping in order to reduce the emotional stress elicited by a challenging situation, rather than directly problem solving and handling the stress at the source [38]. In a situation where individuals feel that they have little control over the situation, they tend to default to avoidant coping [39]. In the current study, maladaptive emotion-focused coping positively predicted perceived stress, which in turn positively predicted psychological distress. This finding was consistent with Compas et al. [40], who suggested that maladaptive emotion-focused coping, such as avoidance coping, has been associated with higher levels of psychological distress and more depressive symptoms. In the current study, adaptive emotion-focused coping negatively predicted perceived stress, which in turn positively predicted psychological distress. This finding suggested that the use of positive emotion-focused coping, such as reappraisal and assigning positive meaning to ordinary events, might help buffer against depressed mood [41] and acute stress [42]. In other words, when the external event is uncontrollable such as the COVID-19 pandemic, one could resort to focusing on positive aspects of one’s emotion in order to reduce perceived stress, leading to lower level of psychological distress.

In general, active problem-focused coping has been related to lower psychological distress [40,43]. Surprisingly, problem-focused coping in the present study positively predicted perceived stress, which in turn positively predicted psychological stress. Given the unprecedented severity and pervasiveness of the impact of the COVID-19 pandemic, most individuals have never encountered such a global event. Even when individuals attempt problem-solving approaches, they have little control over the rapidly changing situations related to the COVID-19, such as public health policy changes, school closures, losing a job due to business closure, having no access to public facilities, and staying in an isolated environment for a prolonged period due to quarantine policy. During the COVID-19 pandemic, even when individuals engage in active coping and problem solving, they cannot change the global situation, and infection rates in different regions continue to fluctuate. It is plausible that the more individuals actively engage in problem solving related to COVID-19, the more they ruminate and worry about the situation, leading to higher levels of perceived stress and higher levels of reported psychological distress. Gan and colleagues [44] examined coping strategies by college students in response to SARS-related stressors. The results showed that participants reported using more avoidant coping with SARS-related stressors that, like COVID-19, were uncontrollable. Such findings suggest that individuals’ coping styles during an unprecedented and sudden COVID-19 pandemic might be different from their coping styles during a typical circumstance, and such difference warrants a differential examination.

There are several limitations of this study that should be noted. First, the study was based on self-reported questionnaires, which might produce potential biases, although our factor analysis results did not indicate a serious common method bias. Second, we recruited undergraduate students exclusively from two universities in Beijing. It is likely that those who responded to the survey were the individuals who wanted to have a voice and were interested in such a research topic. These college students represented highly educated young people in a metropolitan area in China where the societal and public health resources are relatively abundant. Because of this sampling, the findings of this study might not be generalizable to a population with lower educational attainment and more vulnerable occupational status, or to those in other geographical areas. Third, the survey was conducted fully online. Researchers were unable to reach individuals who might not have had internet access during the early stages of the COVID-19 outbreak; such individuals might have been more vulnerable, and might have perceived higher levels of stress associated with COVID-19. Fourth, the present study only focused on the relations between psychological distress, perceived stress, and three types of coping. There might other factors such as subclinical symptoms and emotional difficulties that might affect students’ self-reporting. Such factors should be further explored in future studies.

Future researchers are encouraged to examine a more general population in more diverse regions to capture perceived stress and psychological distress in individuals with different levels of educational attainment, different occupational statuses, and in different regions. Future researchers should consider other measures that could better reflect perceived stress and psychological distress in addition to self-reported questionnaires.

## 5. Conclusions

The present study was to explore the impact of different coping styles on psychological distress during the COVID-19 outbreak among college students in China. The problem-focused and maladaptive emotion-focused coping styles were positively correlated with perceived stress and psychological distress. The maladaptive emotion-focused coping style was negatively correlated with perceived stress and psychological distress. It appears that adaptive, emotion-focused coping could alleviate the mental discomfort associated with the COVID-19 pandemic. The mediating role of perceived stress between the associations of three coping styles and psychological distress found that problem-focused coping and adaptive emotion-focused coping affected psychological distress entirely through the mediation path, in which perceived stress was the mediator. Perceived stress partially mediated the association between maladaptive emotion-focused coping and psychological distress. The findings underscore the importance of perceived stress and provide insights for future intervention. Our findings suggest that mental health service providers might consider providing strategies to help clients reduce their perceived stress. During uncontrollable public health emergencies, strategies and resources that could alleviate one’s perceived stress appear to buffer psychological distress.

## Figures and Tables

**Table 1:** Means, Standard Deviations, and Correlations Among the Variables.

Variables	M	SD	1	2	3	4	5	6	7
1 Gender	–	–	–						
2 Age	19.51	1.52	0.244 ***	–					
3 Problem-focused Coping	3.19	0.76	−0.050	−0.046	–				
4 Adaptive Emotion-focused Coping	2.76	0.53	0.062	0.011	0.581 ***	–			
5 Maladaptive Emotion-focused Coping	1.84	0.65	−0.161 ***	0.005	0.324 ***	−0.028	–		
6 Perceived Stress	2.07	0.73	0.081	0.056	0.154 **	−0.131 **	0.376 ***	–	
7 Psychological Distress	1.99	0.84	−0.044	0.014	0.101 *	−0.185 ***	0.467 ***	0.595 ***	–

Note. Gender (1 = male, 2 = female). * *p* < 0.05. ** *p* < 0.01. *** *p* < 0.001. M = Means; SD = Standard Deviations.

**Table 2:** Bias-corrected bootstrap tests on direct and indirect effects.

Path	Standardized	95% CI	
	(β)	Low	High
Problem-focused Coping → Psychological Distress	0.069	−0.196	0.035
Problem-focused Coping → Perceived Stress → Psychological Distress	0.135	0.056	0.229
Adaptive Emotion-focused Coping → Psychological Distress	−0.143	−0.405	0.077
Adaptive Emotion-focused Coping → Perceived Stress → Psychological Distress	−0.142	−0.240	−0.043
Maladaptive Emotion-focused Coping → Psychological Distress	0.319	0.201	0.438
Maladaptive Emotion-focused Coping → Perceived Stress → Psychological Distress	0.161	0.098	0.223

## Data Availability

We do not provide public access to the data set due to protection of the privacy of the participants. Regarding the details of the data, please contact the corresponding author.

## References

[1] Centers for Disease Control and Prevention (2020). Human Coronavirus Types. https://www.cdc.gov/coronavirus/types.html.

[2] LaFee S. (2021). Novel Coronavirus Circulated Undetected Months before First COVID-19 Cases in Wuhan, China. https://health.ucsd.edu/news/releases/Pages/2021-03-18-novel-coronavirus-circulated-undetected-months-before-first-covid-19-cases-in-wuhan-china.aspx.

[3] Centers for Disease Control and Prevention (2020). Travelers from Countries with Widespread Sustained (Ongoing) Transmission Arriving in the United States. https://www.cdc.gov/coronavirus/2019-ncov/travelers/after-travel-precautions.html.

[4] Downs G. (2020). St. John’s University Asks Students to Vacate Dorms Amid Coronavirus Outbreak. *New York Post*. https://nypost.com/2020/03/10/st-johns-university-asks-students-to-vacate-dorms-amid-coronavirus-outbreak/.

[5] Hess A. (2020). How Coronavirus Dramatically Changed College for over 14 Million Students. CNBC.

[6] Morin M. (2020). NYU Dorms Close, Classes Will Remain Remote for Remainder of Semester. NYU Local.

[7] Watson K. (2020). President Trump Says Coronavirus Crisis Could Last Until July or August. CBS News.

[8] Kerr R. (2020). COVID-19 Brings Uncertainty to Students with Field Work Requirements. The Lantern.

[9] Lazarus R.S., Folkman S. (1984). Stress, Appraisal and Coping.

[10] Lazarus R.S. (1993). Coping theory and research: Past, present, and future. Psychosom. Med..

[11] Endler N.S. (1997). Stress, anxiety and coping: The multidimensional interaction model. Can. Psychol..

[12] Main A., Zhou Q., Ma Y., Luecken L.J., Liu X. (2011). Relations of SARS-related stressors and coping to Chinese college students’ psychological adjustment during the 2003 Beijing SARS epidemic. J. Couns. Psychol..

[13] Folkman S., Lazarus R.S. (1988). Ways of Coping Questionnaire (WAYS) Instrument and Scoring Key.

[14] Suls J., Fletcher B. (1985). The relative efficacy of avoidant and non-avoidant coping strategies: A meta-analysis. Health Psychol..

[15] Tobin L.D., Holroyd K.A., Reynolds R.V., Wigal J.K. (1989). The hierarchical factor structure of the Coping Strategies Inventory. Cogn. Ther. Res..

[16] Kumanova M.V., Karastoyanov G.S. Perceived Stress and Coping Strategies [Paper Presentation]. Proceedings of the Education, Science, Innovation 3rd Annual Conference.

[17] Tian H., Liu Y., Li Y., Wu C.-H., Chen B., Kraemer M.U.G., Li B., Cai J., Xu B., Yang Q. (2020). An investigation of transmission control measures during the first 50 days of the COVID-19 epidemic in China. Science.

[18] Brooks S.K., Webster R.K., Smith L.E., Woodland L., Wessely S., Greenberg N., Rubin G.J. (2020). The psychological impact of quarantine and how to reduce it: Rapid review of the evidence. Lancet.

[19] Holt-Lunstad J., Smith T.B., Layton J.B. (2020). Social relationships and mortality risk: A meta-analytic review. PLoS Med..

[20] Leigh-Hunt N., Bagguley D., Bash K., Turner V., Turnbull S., Valtorta N., Caan W. (2017). An overview of systematic reviews on the public health consequences of social isolation and loneliness. Public Health.

[21] Han K.M., Chang J., Won E., Lee M.S., Ham B.J. (2017). Precarious employment associated with depressive symptoms and suicidal ideation in adult wage workers. J. Affect. Disord..

[22] Mihashi M., Otsubo Y., Yinjuan X., Nagatomi K., Hoshiko M., Ishitake T. (2009). Predictive factors of psychological disorder development during recovery following SARS outbreak. Health Psychol..

[23] Kerr E. (2020). How College Students Manage Coronavirus Stress. U.S. News.

[24] Cohen S., Kamarck T., Mermelstein R. (1983). A global measure of perceived stress. J. Health Soc. Behav..

[25] Han K.S. (2005). Self-efficacy, health promoting behaviors, and symptoms of stress among university students. J. Korean Acad. Nurs..

[26] Rayle A.D., Arredondo P., Kurpius S.E.R. (2011). Educational self-efficacy of college women: Implications for theory, research, and practice. J. Couns. Dev..

[27] Carver C.S. (1997). You want to measure coping but your protocol’s too long: Consider the Brief COPE. Int. J. Behav. Med..

[28] Yeung D.Y.-L., Fung H.H. (2007). Age differences in coping and emotional responses toward SARS: A longitudinal study of Hong Kong Chinese. Aging Ment. Health.

[29] Antony M.M., Bieling P.J., Cox B.J., Enns M.W., Swinson R.P. (1998). Psychometric properties of the 42-item and 21-item versions of the Depression Anxiety Stress Scales in clinical groups and a community sample. Psychol. Assess..

[30] Zhou C.-C., Chu J., Wang T., Peng Q.-Q., He J.-J., Zheng W.-G. (2008). Reliability and validity of 10-item Kessler Scale (K10) Chinese version in evaluation of mental health status of Chinese population. Chin. J. Clin. Psychol..

[31] Kessler R.C., Andrews G., Colpe L.J., Hiripi E., Mroczek D.K., Normand S.L.T., Walters E.E., Zaslavsky A.M. (2002). Short screening scales to monitor population prevalences and trends in nonspecific psychological distress. Psychol. Med..

[32] Bandura A. (1997). Self-Efficacy: The Exercise of Control.

[33] Chu D.K., Akl E.A., Duda S., Solo K., Yaacoub S., Schunemann H.J. (2020). Physical distancing, face masks, and eye protection to prevent person-to-person transmission of SARS-CoV-2 and COVID-19: A systematic review and meta-analysis. Lancet.

[34] Lorant V., Smith P., Van den Broeck K., Nicaise P. (2021). Psychological distress associated with the COVID-19 pandemic and suppression measures during the first wave in Belgium. BMC Psychiatry.

[35] Lazarus R.S. (1990). Theory-based stress measurement. Psychol. Inq..

[36] Cannon W.B. (1932). Effects of Strong Emotions.

[37] Melamed S., Shirom A., Toker S., Shapira I. (2006). Burnout and risk of type 2 diabetes: A prospective study of apparently health employed persons. Psychosom. Med..

[38] Carver C.S., Scheier M.F., Weintraub J.K. (1989). Assessing coping strategies: A theoretically based approach. J. Personal. Soc. Psychol..

[39] Folkman S., Lazarus R.S. (1980). An analysis of coping in a middle-aged community sample. J. Health Soc. Behav..

[40] Compas B.E., Connor-Smith J.K., Saltzman H., Thomsen A.H., Wadsworth M.E. (2001). Coping with stress during childhood and adolescence: Problems, progress, and potential in theory and research. Psychol. Bull..

[41] Davis C.G., Nolen-Hoeksema S., Larson J. (1998). Making sense of loss and benefiting from the experience: Two construals of meaning. J. Personal. Soc. Psychol..

[42] Folkman S., Moskowitz J.T. (2000). Positive affect and the other side of coping. Am. Psychol..

[43] Snow-Turek A.L., Norris M.P., Tan G. (1996). Active and passive coping strategies in chronic pain patients. Pain.

[44] Gan Y., Liu Y., Zhang Y. (2004). Flexible coping responses to severe acute respiratory syndrome-related and daily life stressful events. Asian J. Soc. Psychol..

